# Editorial: can China master the guideline challenge?

**DOI:** 10.1186/1478-4505-11-1

**Published:** 2013-01-09

**Authors:** Kehu Yang, Yaolong Chen, Youping Li, Holger J Schünemann

**Affiliations:** 1 Evidence-Based Medicine Center, School of Basic Medical Sciences, Lanzhou University, Lanzhou, China; 2 Chinese Cochrane Center, Chengdu, China; 3 Departments of Clinical Epidemiology & Biostatistics and of Medicine, McMaster University Health Sciences Centre, Room 2C16, 1280 Main Street West, Hamilton, ON, L8S 4K1, Canada

## Abstract

China is experiencing increased health care use and expenditures, without sufficient controls to ensure quality and value. Transparent, cost-conscious and patient-centered guidelines based on the best available evidence could help establishing these quality and practice measures.

We examined how guidelines could support the Chinese health reform. Specifically, we summarized the current state of the art and related challenges in guideline development and explored possible solutions in the context of the Chinese health reform.

China currently lacks capacity for evidence-based guideline development and coordination by a central agency. Most Chinese guideline users rely on recommendations developed by professional groups that lack demonstration of transparency (including conflict of interest management and evidence synthesis) and quality. These deficiencies appear larger than in other regions of the world. In addition, misperceptions about the role of guidelines in *assisting practitioners* as opposed to *providing rules* requiring adherence, and a perception that traditional Chinese medicine (TCM) cannot be appropriately incorporated in guidelines are present.

China’s capacity could be strengthened by a central guideline agency to provide or coordinate evidence synthesis for guideline development and to oversee the work of guideline developers. China can build on what is known and work with the international community to develop methods to meet the challenges of evidence-based guideline development.

## China’s health care challenges in the context of guideline development

In his opening remarks to an international symposium on guideline development, Prof. YinDakui, former Chinese Vice Minister of Health and now president of the Chinese Association of Medical Doctors, stated that “all medical practitioners in China should learn about and apply high-quality evidence-based guidelines to practice in order to make contributions to the health of people in China as well as the world”. But how can this vision become reality? The authors of this article organized an international conference and brought together leading scientists in evidence synthesis and Chinese health policy to examine how guidelines could support the Chinese health reform. In this article, together with the conference speakers, we summarize the current state of the art and related challenges in guideline development and explored possible solutions in Lanzhou, China, on May 28 and 29, 2012, specifically in the context of the Chinese health reform.

China is experiencing increased health care use and expenditures, without sufficient controls to ensure quality and value. For example, between 2003 and 2011, the insurance coverage increased from 30% to 96% and the average share of inpatient costs reimbursed from insurance increased from 14 to 47. There has been concern about rising of cost and the Ministry of Health of China called for a control of medical expenses in 2012[1]. Measures and instruments of care delivery such as rates of caesarian sections, access to essential medicines and independent evaluation of health care services are important but insufficient as indicators to monitor quantity and quality of care provided. For example, WHO reported that China has the world's highest rate of caesarian sections and that one quarter of them were not medically necessary [2,3]. However, these measures do not cover the entire spectrum of care and allowing care providers to concentrate on a few selected measures rather than establishing best practice broadly could mean ignoring other areas of health care delivery, in particular in large countries like China. Transparent, cost-conscious and patient-centered guidelines based on the best available evidence could help establishing these quality and practice measures and contain resource expenditure. In fact, guidelines are becoming an important part of national health care reforms in China because they may reduce health care inequities and improve the quality of medical care.

## Who might develop guidelines in China?

Ideally, guidance in China would be provided by national agencies in collaboration with other stakeholders, including practitioners, public health experts, the public. Western countries can provide examples, e.g. the National Institute of Health and Clinical Excellence (NICE) in the UK. However, given the Chinese context of TCM, local variations in the implementation of the health reform, and the massive size of the population, adaptation of such models is needed that may require close collaboration between central and regional government, academia, professional associations and local health care leaders while ensuring the highest possible level of transparency. Despite these challenges, China can take advantage of the work that has been performed by many guideline developers, team up with these organizations and take advantage of organizations like the guideline international network (GIN).

## State of the art of guideline development

According to the World Health Organisation’s definition, appropriately developed guidelines, based on the best available evidence, should assist providers and recipients of health care and other stakeholders to make informed decisions. Recommendations [in guidelines] may relate to clinical interventions, public health activities, or government policies [4]. International standards for the development of such guidelines are available and they agree on many key points such as question definition, using credible and valid systematic reviews, grading of evidence and strength of recommendations and management of conflict of interest [5-7]. These standards have become more stringent over time, because of research on new methods and conceptual work in guideline development, for example in the area of managing conflict of interest. Despite the progress in defining criteria for trustworthy guideline development, gaps in research around guideline group composition and processes, determining and implementing criteria for moving from evidence to recommendations and developing recommendations about diagnostic tests and strategies are apparent. Thus, China can build on what is known and work with the international community to develop methods to meet the challenges of evidence-based guideline development.

## Guidelines in China: status quo

China currently lacks capacity for evidence-based guideline development and coordination by a central agency. Most Chinese guideline users rely on recommendations developed by professional groups that lack demonstration of transparency (including conflict of interest management and evidence synthesis) and quality. These deficiencies appear larger than in other regions of the world [8,9]. As of June 2012, the Guidelines International Network (G-I- N) database lists more than 7200 guidelines (http://www.g-i-n.net/library) and the National Guideline Clearinghouse (NGC) contains about 2300 (http://www.guideline.gov). However, no guideline from mainland of China is documented in either of these databases. There is no specific organizational structure that supports clinical trials, systematic reviews and guideline development. In addition, conference attendees witnessed misperceptions about the role of guidelines in *assisting practitioners* as opposed to *providing rules* requiring adherence, and a perception that traditional Chinese medicine (TCM) cannot be appropriately incorporated in guidelines. These concerns stem from the belief that TCM is based on centuries of observations and the holistic concept of TCM that cannot be reevaluated or appropriately assessed in research studies that reduce bias. Proper research designs could however be employed to either summarize what is known or plan studies that avoid bias.

## Possible solutions to the challenges

China’s capacity could be strengthened by a central guideline agency to provide or coordinate evidence synthesis for guideline development and to oversee the work of guideline developers. A central health technology assessment unit, a China NICE, the Chinese Cochrane Center or the Chinese GRADE center could support or fulfill such a role [10]. If progress can be made, Chinese guideline developers can adopt approaches that make guidelines trustworthy and they can conduct research on best guideline development practices. Registration and certification for guideline developers to adhere to minimal standards in evidence assessment and transparent development and reporting of recommendations [11,12], and monitoring with tools such as AGREE [13] are steps that can avoid duplication and ensure credibility of guidelines. China can share evidence (both data and evidence syntheses) and support capacity building in guideline development through international collaborations. But equally important are national collaborations, between guideline developers, health policy makers and media to publish guidelines that are developed to allow user-friendly products for clinicians, patients, and policy makers. Education about the intended purpose and use of guidelines focusing on patient centered care will be central in moving forward. Finally, public access at all levels to guidelines through central (financial) support will allow for greater guideline availability.

## Conclusions

China has an excellent opportunity to build on experience of international guideline development efforts, avoid the mistakes they made and set an example for other emerging countries. We advocate that for all guidelines, the development process should be transparent leading to actionable recommendations and advocate for patients’ interests by acknowledging their preferences and values. It could mean the beginning of a new era for health care in China.

## Authors’ information

Members of the Lanzhou International Guideline Symposium:

Sarah L Barber, World Health Organization, Beijing, China

Jan Brozek, Department of Clinical Epidemiology and Biostatistics, McMaster University, Hamilton, Canada

Françoise Cluzeau, National Institute for Health & Clinical Excellence International, London, UK

Marina Davoli, Dipartimento Epidemiologia del S.S.R. - ASL RME Regione Lazio, Rome, Italy

Roman Dong Xu, Beijing Office of China Medical Board, Beijing, China

Yngve Falck-Ytter, Case Western Reserve University, Case and VA Medical Center, Cleveland, Ohio, USA

Signe Flottorp, Norwegian Knowledge Centre for the Health Services, University of Bergen, Bergen, Norway

Laragh Gollogly, World Health Organization, Geneva, Switzerland

Anne Lethaby, Research Consultant, Auckland, New Zealand

Susan L. Norris, Oregon Health & Science University, Portland, OR, USA

Nancy Santesso, Department of Clinical Epidemiology and Biostatistics, McMaster University, Hamilton, Canada

Rob J.P.M. Scholten, Deparment of Clinical Epidemiology, Biostatistics and Bioinformatics, Academic Medical Center, Amsterdam, The Netherlands

Kun Zhao, China National Health Development and Research Center, Division of Health Policy Evaluation and Technology Assessment, Beijing, China
